# The influence of listener experience, measurement scale and speech task on the reliability of auditory-perceptual evaluation of vocal quality

**DOI:** 10.1590/2317-1782/20232023175

**Published:** 2024-04-15

**Authors:** Jônatas do Nascimento Alves, Anna Alice Figueiredo de Almeida, Rosiane Yamasaki, Leonardo Wanderley Lopes

**Affiliations:** 1 Universidade Federal da Paraíba - UFPB - João Pessoa (PB), Brasil.; 2 Universidade Federal de São Paulo - UNIFESP - São Paulo (SP), Brasil.

**Keywords:** Voice, Auditory-perceptual Analysis, Severity of voice Disorder, Vocal Quality, Voice Disorders, Reliability

## Abstract

**Purpose:**

To assess the influence of the listener experience, measurement scales and the type of speech task on the auditory-perceptual evaluation of the overall severity (OS) of voice deviation and the predominant type of voice (rough, breathy or strain).

**Methods:**

22 listeners, divided into four groups participated in the study: speech-language pathologist specialized in voice (SLP-V), SLP non specialized in voice (SLP-NV), graduate students with auditory-perceptual analysis training (GS-T), and graduate students without auditory-perceptual analysis training (GS-U). The subjects rated the OS of voice deviation and the predominant type of voice of 44 voices by visual analog scale (VAS) and the numerical scale (score “G” from GRBAS), corresponding to six speech tasks such as sustained vowel /a/ and /ɛ/, sentences, number counting, running speech, and all five previous tasks together.

**Results:**

Sentences obtained the best interrater reliability in each group, using both VAS and GRBAS. SLP-NV group demonstrated the best interrater reliability in OS judgment in different speech tasks using VAS or GRBAS. Sustained vowel (/a/ and /ɛ/) and running speech obtained the best interrater reliability among the groups of listeners in judging the predominant vocal quality. GS-T group got the best result of interrater reliability in judging the predominant vocal quality.

**Conclusion:**

The time of experience in the auditory-perceptual judgment of the voice, the type of training to which they were submitted, and the type of speech task influence the reliability of the auditory-perceptual evaluation of vocal quality.

## INTRODUCTION

Voice quality is a perceptual phenomenon by nature^(1,2)^ and can be considered the listener’s global auditory impression of the speaker’s voice^(3,4)^. Thus, voice quality can be understood as an interaction between the listener and the speaker’s voice signal in which the listener takes advantage of the acoustic information available in that sound signal to achieve a specific perceptual goal^(5-10)^.

Auditory-perceptual evaluation of voice can be considered an effective method to describe an individual’s vocal profile, characterize voice quality, and quantify vocal deviation^(11)^. It provides data on the characterization of vocal deviation intensity (extent of this deviation) and on the predominant voice quality (type of vocal deviation, such as roughness or breathiness)^(4,12,13)^.

Although the are criticisms about its subjectivity, auditory-perceptual evaluation is traditionally used in the clinical context of speech-language pathology and is considered the main reference standard for voice analysis^(11,14)^. It can be influenced by some factors, such as listener experience^(1,12,15-17)^, type of rated speech task^(4,13,18,19)^, and the rating scale^(1,6,20,21)^.

Listener experience may affect the auditory-perceptual evaluation of voice quality^(1,12,17,22)^. Some research indicates an increase in reliability among more experienced listeners^(1,12)^, whereas some shows no differences between experienced and inexperienced listeners^(23)^. Thus, the relationship between experience and auditory-perceptual analysis is not yet properly defined, especially when a study includes listeners with different levels of experience^(16)^. Therefore, the impact of this factor on voice quality evaluation must be assessed^(12,24)^.

The evaluated speech task may also influence the perceptual evaluation of voice^(6,13,25-27)^. In general, auditory-perceptual ratings of phonated vowels may be more reliable than ratings of connected speech, but there is research that suggest this trend may be reversed^(28)^. In addition, some experiments show no significant differences in the evaluation regardless of the speech task chosen^(29,30)^. These inconsistent findings highlight the importance of further studies with experimental designs addressing different speech tasks to more comprehensively understand the influence of this factor on the auditory-perceptual evaluation of voice quality^(19)^.

The measurement scales used in the auditory-perceptual evaluation of voice also vary. Several studies have compared different scales in the perceptual analysis of voice, including the visual analog scale (VAS) and the numerical scale (NS)^(6,21,31-35)^. In general, the results indicate that the VAS has better interrater agreement in perceptual evaluation^(21,31-33)^ than the NS. In addition, the VAS is more sensitive to small differences in vocal deviation^(21,34,35)^.

Considering the various factors that influence the auditory-perceptual evaluation of voice and its relevance to the clinical assessment of voice, researchers strive to develop tools to reduce the variability and inconsistencies of this evaluation in order to improve interrater reliability^(6,27)^. These include Grade, Roughness, Breathiness, Asthenia, Strain from GRBAS scale^(36,37)^ and Consensus Auditory-Perceptual Evaluation of Voice Protocol (CAPE-V)^(38)^. These protocols seek to determine the speech tasks to be analyzed, as well as the measurement scale used by the raters in the judgment^(25,27)^.

Clinicians and researchers continually seek to improve the evidence base in studies involving voice disorders in order to standardize the procedures and protocols used for clinical voice evaluation^(14,27)^. Based on the above, the results of the present study may help to understand the influence of speech task on rater reliability, to define the tasks with the highest degree of agreement, and to broaden the understanding of the influence of listener experience and measurements scales on the auditory-perceptual analysis of voice. The data generated by this study may help to standardize voice quality evaluation procedures, which may improve communication between clinicians and researchers in comparing data, thereby bolstering evidence on voice disorders^(27)^. Therefore, our objective is to assess the influence of the listener experience, measurement scales and the type of speech task on the auditory-perceptual evaluation of the overall severity (OS) of voice deviation and the predominant type of voice (rough, breathy or strain).

## METHODS

### Study design

This is a descriptive, cross-sectional, and observational study, previously evaluated and approved by the Research Ethics Committee from the University of origin, under opinion No. 5.174.946/21. This study was conducted at the Voice Laboratory.

### Participants

The study subjects were 22 listeners divided into four groups by experience in auditory-perceptual analysis, as outlined in Chart 1. For the present research, we used the criteria by Hill et al.^(38)^ to define the groups that will be investigated. These authors describe four levels of training: pre-novice, which generally includes individuals (undergraduate students) in the basic cycle of training before professional disciplines and with a low degree of autonomy related to the profession; novice, undergraduate students studying practical and professional disciplines or at the end of the undergraduate course, with an intermediate degree of autonomy in the profession; entry-level, professionals who have up to five years from graduation, with an intermediate degree of autonomy in the profession; intermediate, corresponds to professionals between 5 and 10 years after graduation, with moderate autonomy in the profession; and senior, professionals with more than ten years of experience, in general, in a specific area of expertise, with full professional autonomy.

**Chart 1:** Description of the stratification of listeners by experience

*Stratification of listeners by experience*	
*Group 1 (n=7)*	*Graduate students without auditory-perceptual training (GS-U)*
*Group 2 (n=7)*	*Graduate students with auditory-perceptual training (GS-T)*
*Group 3 (n=4)*	*Speech-language pathologists (SLP) non specialized in voice (SLP-NV)*
*Group 4 (n=4)*	*SLP specialized in voice, with more than 10 years of experience in auditory-perceptual evaluation of voice quality (SLP-V)*

Specifically, we considered the pre-novice, novice, entry-level, and senior groups for this research. Individuals in the intermediate group were not recruited since, in Brazil, such professionals who work with voice have already completed some specialization in the area. In addition, we used the criterion of having previously performed auditory-perceptual training and having specialization in voice for the composition of the groups. Additionally, we have renamed the groups to make them better suited to the interests of this research. Thus, the pre-novice group became known as “undergraduate students without auditory-perceptual training (GS-U)”; the novice group became known as “undergraduate students with auditory-perceptual training”; the entry-level became known as “Speech-language pathologists (SLP) non-specialized in voice (SLP-NV)”; and the senior group was called “SLP specialized in voice (SLP-V)”.

For the listeners of GS-U (corresponds to the pre-novice group), the following eligibility criteria were used: Speech-Language Pathology students at an undergraduate program without self-reported neurological or auditory deficit preventing them from signing the informed consent form, excluding those with prior training in auditory-perceptual analysis of voice, who have taken specific theoretical-practical courses in the voice area or have carried out research and extension activities in the voice area. Thus, this group had only students in the first three semesters of the undergraduate degree in Speech-Language Pathology, totaling seven volunteers.

To recruit the listeners of GS-U, we contacted the Council of the Undergraduate Degree in Speech-Language Pathology at University of origin, which provided access to the students. Thus, to recruit the GS-U, we contacted those in their third semester of the degree, which consisted of students without prior training in auditory-perceptual evaluation, as stated above. Fourteen students showed interest in participating in the study. However, four were excluded because they were involved in research and extension activities in the voice area (which included auditory-perceptual training) and 3 were excluded due to unavailability of schedule to participate in the research. Of the 14 students enrolled in the degree, seven were participating in the study sessions.

GS-T also included students enrolled in the undergraduate degree in Speech-Language Pathology at an undergraduate program without any self-reported neurological or auditory deficit preventing them from signing the informed consent form, but consisting only of students who had already been trained in auditory-perceptual analysis of voice and who had gained experience in internships or university extension programs related to the field of voice; accordingly, the group consisted of students at more advanced stages of the degree (completed 6 to 7 semesters), totaling seven volunteers. To recruit the listeners of GS-T, we contacted the Council of the Undergraduate Degree in Speech-Language Pathology at University of origin, which provided access to the students. For GS-T, undergraduate students with experience, we contacted the university extension program in voice screening of the Speech-Language Pathology School Clinic at the same undergraduate program. The students of this school clinic had completed the most advanced semesters of the degree (6^th^ and 7^th^), and all 7 students enrolled in the university extension program were interested in and available to participate in the study.

SLP-NV consisted of general SLPs who were not specialized in voice and who had less than five years of experience in auditory-perceptual analysis of voice quality. All participants of this group had graduated from the same program of the GS-U and GS-T, and received the same auditory-perceptual analysis training as the students of GS-U and GS-T during their undergraduate degree. In addition, the participants of this group should work weekly in the voice area, full or part time.

SLP-V consisted of four specialized SLPs with at least 10 years of experience in auditory-perceptual analysis of voice quality. These SLPs should work full-time in the voice area throughout their 10-year career. Importantly, this group was homogeneous in experience time but heterogeneous in experience type. Their specializations and training in auditory-perceptual evaluation included training in numerical scales (GRBAS), visual analog scales (CAPE-V), and vocal profile analysis schemes (VPAS). The listeners also differed qualitatively in experience type: some listeners were more experienced in assessing behavioral voice disorders, whereas other listeners were more experienced in evaluating voices of patients with sequelae of head and neck cancer and neurogenic dysphonia, or were more experienced in assessing voice professionals. All of these students should have completed a mandatory discipline of the undergraduate course, where 12 hours of auditory-perceptual training are carried out.

For SLP-NV, general SLPs with less than 5 years of experience, we directly contacted these healthcare professionals. Approximately 10 were contacted, and 4 showed interest in and were available to participate in the study. All subjects of this group had graduated from the same higher education institution from which the subjects of groups 1 and 2 were recruited. In this way, we guarantee that the participants of the GS-T and SLP-NV groups have gone through the same auditory-perceptual training (both in terms of duration - 12 hours, and in terms of training structure) during their undergraduate course. In addition, all SLPs recruited for this group did not exclusively care for patients in the voice area, working with patients with other communication and swallowing disorders.

SLP-V, specialized SLPs with more than 10 years of experience, consists of four graduate and undergraduate professors about voice contents.

### Procedures

#### Sample preparation

The stimuli used for auditory-perceptual analysis of voice quality were retrieved from the corpus of the database of the Voice Laboratory where this study was realized. This database consisted of voice recordings of patients who voluntarily seek the help of this laboratory for voice quality evaluation.

In the voice evaluation routine of this Laboratory, the procedures are explained to the patients, and the collected data are used for research purposes once they sign an informed consent form. The voice evaluation procedures performed at this Laboratory include administration of a brief clinical history questionnaire and voice self-assessment protocols, voice recording, and laryngeal visual examination. In the present study, we used only data related to the voice signal of the patients. Follow, we will describe the recording conditions of the speech samples.

Voice samples were recorded in a soundproof recording booth with background noise lower than 20 dB sound pressure level (SPL) at a 44,000-Hz sampling rate and 16 bits/sample, using the software Fonoview, version 4.5 (Pato Branco, Paraná, Brazil ), a Dell all-in-one desktop computer (Eldorado do Sul, Rio Grande do Sul, Brazil), and a Sennheiser E-835 unidirectional cardioid microphone placed on a stand and plugged into a Behringer preamplifier (U-Phoria UMC 204, Willich, Germany).

At the time of the recording, the patients are asked to complete different speech tasks. For this study, we accessed only the following tasks in the database: (1) sustained vowel /a/ at a self-selected comfortable vocal intensity and frequency; (2) sustained vowel /ɛ/ at a self-selected comfortable vocal intensity and frequency; (3) counting from 1 to 10 at the typical speech rate and vocal intensity; (4) phonetically balanced CAPE-V sentences (“*Érica tomou suco de pêra e amora*” [“Erica drank pear and blackberry juice”], “*Sônia sabe sambar sozinha*” [“Sonia knows how to dance the samba alone”], “*Olha lá o avião azul*” [“Look at the blue plane”], “*Agora é hora de acabar*” [“Time to wrap up”], “*Minha mãe namorou um anjo*” [“My mother dated an angel”], “*Papai trouxe pipoca quente*” [“Daddy brought hot popcorn”]), validated for Brazilian Portuguese; (5) a running speech sample, which was the patient’s own account of their motivation to seek care in the clinic. The rational for choosing these tasks is described in Chart 2.

**Chart 2:** Description of the rationale for choosing each speech task used in the study

**TASK**	**RATIONALE**
Sustained vowel /a/	Sustained vowels show less variation in time and involve a more stable relationship between glottal and supraglottal adjustments. In addition, they are not affected by the phonetic context or prosodic variations^(39)^.
	The vowel /a/ is the most commonly used in studies involving perceptual judgment of voice quality (VQ) internationally^(18)^.
Sustained vowel /ɛ/	Sustained vowels show less variation in time and a more stable relationship between glottal and supraglottal adjustments. In addition, they are not affected by the phonetic context or prosodic variations^(39)^.
	Considered the most neutral and central vowel of Brazilian Portuguese (BR-PT), /ɛ/ is the vowel most commonly used to evaluate the VQ in Brazil^(40)^.
Counting	Because counting is a connected speech task, it can change the vocal tract configuration, contains key perceptual clues to the definition of vocal deviation in normal voice use^(41-43)^, and may show good interrater reliability^(18)^.
CAPE-V sentences	Six phonetically balanced sentences for evaluating voice quality in different phonetic contexts^(25,38)^.
	● *Érica tomou suco de pêra e amora* [Erica drank pear and blackberry juice] (Covers all vowels of BR-PT)
	● *Sônia sabe sambar sozinha* [Sonia knows how to dance the samba alone] (focuses on the phoneme /S/; unvoiced/voiced phoneme transition)
	● *Olha lá o avião azul* [Look at the blue plane] (fully voiced sentence)
	● *Agora é hora de acabar* [Time to wrap up] (Vowel-initial words that may trigger sudden vocal attacks)
	● *Minha mãe namorou um anjo* [My mother dated an angel] (Incorporates nasal sounds)
	● *Papai trouxe pipoca quente* [Daddy brought hot popcorn] (Sentence with several unreleased stops)
Running speech	Of all speech tasks proposed in this study, running speech is the closest to everyday speech^(13,25)^.
All five previous tasks together	The use of different speech tasks may enable a more global perception of vocal deviation.

**Figure 1:**
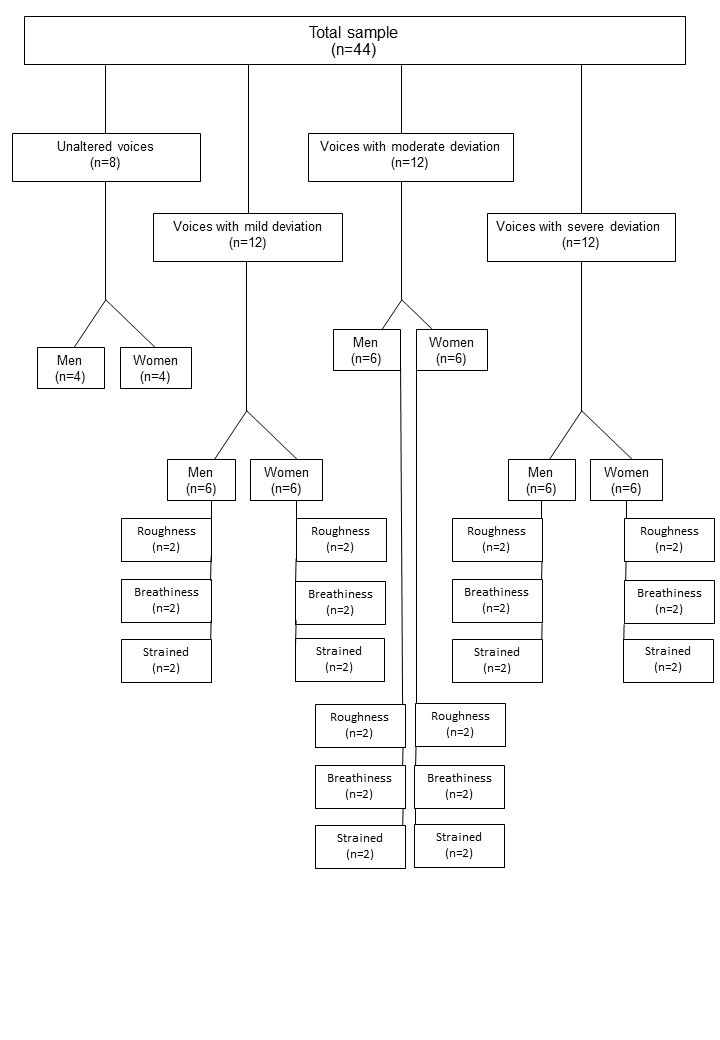
Flowchart of the quantitative distribution of healthy and deviated voices

Considering the time required to perform the auditory-perceptual analysis and the representativeness of different degrees and types of vocal deviation, 8 samples of healthy voices (4 male and 4 female), 12 rough voices, 12 breathy voices, and 12 strained voices, all with balanced representativeness in terms of degree (mild, moderate, and severe) and gender (Figure 1), were selected for this study. The three types of predominant voices (rough, breath and strain) addressed in this study were chosen because they are the most commonly found among individuals with voice disorders^(2,44)^.

**Caption:** SVE (sustained vowel /ɛ/); SVA (sustained vowel /a/); SENT (sentences); CTG (counting); RUN (running speech); GLOB (interconnected tasks). Group 1 (undergraduate students in Speech-Language Pathology without experience); Group 2 (undergraduate students with experience); Group 3 (general speech-language pathologists); Group 4 (voice specialized speech-language pathologists)

All voice recordings in the database had been previously subjected to auditory-perceptual evaluation by a SLP specialized in voice and with more than 10 years of experience. The conditions under which this auditory-perceptual analysis recorded in the database was performed is described next.

Initially, the SLP was trained with 16 anchor stimuli (sustained vowel /ɛ/), made up of four samples from vocally healthy subjects, four samples from individuals with mild-to-moderate vocal deviation, four samples from individuals with moderate vocal deviation, and four samples from individuals with severe vocal deviation. The SLP was asked to listen to the anchor stimuli immediately before analyzing the voices of this study. All samples selected for training had been previously analyzed by SLP experienced in voice analysis and routinely used for auditory-perceptual training and as anchor stimuli at the study Laboratory.

For the auditory-perceptual analysis of these voices, the visual analog scale (VAS), ranging from 0 to 100 mm, was used to assess the overall severity of vocal deviation (OS). The listener was informed that marks closer to 0 represented the most socially acceptable voices produced most naturally, with least effort, noise or instability. Conversely, marks closer to 100 represented the least socially acceptable voices produced with most perceived effort, noise and instability. The listener was also instructed that roughness corresponded to the presence of vibratory irregularity, breathiness was related to audible air escape in phonation, and strain corresponded to perceived vocal effort throughout phonation. After marking the OS in the VAS, the listener should identify predominant vocal deviation (rough, breathy, strain or no predominant vocal deviation). We excluded from this analysis all voices that were identified as deviated, but without predominant vocal quality perceived auditory by the listener. The rating reliability of this listener was assessed using Cohen’s kappa coefficient; a coefficient of 0.80 was calculated, which indicates good intra-reliability.

Thus, after retrieving the results of the auditory-perceptual evaluation of this listener from the database, the first 44 samples, whose voice signals had received the ratings shown in Figure 1, were selected.

For this study we used six speech tasks from each these 44 samples, including: sustained vowel /a/, sustained vowel /ɛ/, sentences, number counting, running speech, and all five previous tasks together. Therefore, we had a total of 220 speech samples.

Shortly after the selection, the samples were edited in the software SoundForge, version 10.0. For vowels, the first and last two seconds of each vowel phonation were eliminated, due to their higher irregularity, keeping a three-second sample for each phonation and including only the subjects who sustained the vowels for this length of time, as mentioned above. The intervals between CAPE-V sentences were edited and standardized, maintaining 1 second between sentences, and this task lasted from 12 to 14 seconds. For number counting, the audio files were edited only when the intervals were longer than 1 seconds during the count. For the running speech task, an interval of 8 to 10 seconds was chosen.

All stimuli were subjected to normalization, performed in the “normalize” control of SoundForge, in peaklevel mode, to standardize the audio output from -6 to 6 dB. The aim of this procedure was to avoid the effect of vocal intensity on the auditory-perceptual judgment of voice quality. Last, all speech tasks of the same patient were edited into a single file in .wav format, named “all tasks”.

After selecting and properly editing the samples, the voices were randomly organized in PowerPoint to present them to the listeners for auditory-perceptual evaluation. The voices were divided for presentation into two blocks: (1) five speech tasks (sustained vowel /a/, sustained vowel /ɛ/, sentences, number counting, and running speech) edited individually; (2) all speech tasks of each subject presented in a single audio file (“all tasks” file).

The first block was methodologically divided into five subgroups according to individual tasks, that is, each subgroup had only one specific speech task. Thus, the listeners listened to the 44 audio files of each individual task, totaling 220 samples. The order of presentation of these subgroups for individual speech tasks was defined by draw using the online tool at sorteador.com.br. Thus, the final order of presentation was vowel /a/, vowel /ɛ/, CAPE-V sentences, number counting, and running speech. The order of presentation of the voices in each subgroup was also randomized, by draw, using the software Radom MixTaper Maker. Thus, the subject “Voice 1” of the vowel /a/ group was not necessarily the same as that of the vowel /ɛ/ group, and so forth.

The second block, named “all tasks”, consisted of a total set of 44 samples, containing the five speech tasks of each subject in a single .wav file. In this block, the order of sample presentation was randomized using Radom MixTaper Maker.

In this study, 22 listeners, subdivided into four groups (GS-U, GS-T, SLP-NV, and SLP-V), participated in the auditory-perceptual judgment. To collect data for the auditory-perceptual analysis, two scales were used to assess the OS, VAS and numerical scale GRBAS, in addition to the item “predominance”, referring to the predominant type of voice during phonation (rough, breathy, strained, or unidentifiable predominance).

The visual analog scale (VAS) from 0 to 100 mm, for measuring vocal deviation intensity, was also used because the aim of this study was to investigate the association between the rating scale and the reliability of the listeners. In the VAS, 0 represents the lowest vocal deviation intensity and 100 the highest vocal deviation intensity. To classify the patients regarding the presence of vocal deviation, a cutoff point of 35.5 mm was used^(21)^, with the following ranges: from 0 to 35.5 for individuals without changes; from 35.6 to 50.5 for individuals with mild vocal deviations; from 50.6 to 90.5 for subjects with moderate changes; and from 90.5 to 100 for individuals with severe vocal deviation^(21)^.

GRBAS^(37)^ has five parameters for voice quality assessment: G - grade, R - roughness, B - breathiness, A - asthenia, S - strain. This is a four-point numerical scale, considering 0: normal/healthy voice; 1: mild vocal deviation; 2: moderate vocal deviation; and 3 severe vocal deviation. Thus, because the aim of this study was to investigate the reliability of raters in assessing the OS of vocal deviation, only the parameter “G” of the GRBAS was used.

### Test application

The auditory-perceptual analysis was divided into two sessions to minimize effects of the raters’ auditory memory^(13,45)^. There was a 7-day interval between the evaluation of block 1 (individual tasks), termed “session 1”, and the evaluation of block 2 (all tasks), termed “session 2”. Both sessions occurred in a silent environment, with background noise lower than 50 dB SPL, and lasted, on average, 60 minutes.

In session 1, the listeners filled out an identification form, with data on experience time, university major, and whether the listener was an undergraduate student and a speech-language pathologist specialized in voice. They were informed about the aims of the study and, if available and willing to participate, were asked to fill out the informed consent form. Thus, all listeners were told that they would listen to voices of individuals in specific speech tasks and that, in each presentation, they should score the predominant voice quality, OS on the VAS and G in GRBAS.

The listeners were instructed to identify the predominant type of voice. For this purpose, the category “healthy voice” was used when the voice showed no auditorily perceived vocal deviation. When the voices were deemed to deviate from auditorily perceived voice quality, the listeners categorized them as predominantly rough, breathy, or strained. The category “without identifiable predominance” was added for when it was deemed impossible to identify the predominant voice quality. Healthy voices corresponded to a socially acceptable voice, naturally produced without effort, noise, or instability during phonation^(21)^. Roughness was related to the presence of vibratory irregularity. Breathiness corresponded to the presence of unvoiced transglottal air (audible). Strain corresponded to perceived vocal effort throughout phonation^(21,25)^.

Subsequently, we used a set of anchor stimuli containing samples of healthy male and female voices and with different degrees and types of vocal deviation. All anchor stimuli used in this study were retrieved from the database of the Voice Laboratory, and these samples had already been previously analyzed by SLPs experienced in voice analysis and routinely used for auditory-perceptual training and as anchor stimuli at this Laboratory.

These stimuli were presented to the listeners using a speaker, which was available whenever the listener any doubt about the reference parameter throughout the auditory-perceptual evaluation. To complete the orientation, the listeners were specifically instructed about how to score the predominant voice quality, how to score OS on the VAS, and how to score G in GRBAS.

After this initial period of orientation, a speech sample from an individual excluded from the study samples was used to define the vocal intensity of stimulus presentation to the listeners. The stimulus was presented using a Nipponic Cr 616 loudspeaker, in open field, at a volume loud enough and comfortable for the listeners to rate the stimuli.

In this session 1, 220 samples were presented in subsets of 44 stimuli, with a 5-minute interval between the presentations of subsets. This procedure aimed to minimize possible attention lapses and hearing fatigue^(13)^. For each presented stimulus, the listeners scored the predominant voice quality and vocal deviation intensity measured using the VAS and GRBAS, as previously described. The stimuli could be repeated a maximum of three times if requested by the listeners.

### Data analysis

To analyze rater reliability, the following tests were used: intraclass correlation coefficient (ICC), Cohen’s kappa coefficient (kappa), and first-order agreement coefficient (AC1). ICC is one of the most widely used statistical tools for assessing measurement reliability and is the most commonly used agreement measure for continuous data^(1,46,47)^; therefore, in this study, this test was used only for interrater reliability from each group, with data involving the VAS because this is a continuous scale. The degree of reliability, based on the ICC, ranges from low to excellent, according to the following values: lower than 0.50, low reliability; from 0.50 to 0.75, moderate reliability, from 0.75 to 0.90; good reliability; and higher than 0.90, excellent reliability^(48)^.

The kappa measure was used to analyze the interrater reliability from each group of the GRBAS G domain and the predominant voice quality. The degree of agreement, based on the kappa coefficient, ranges from slight to almost perfect agreement, according to the following values: from 0 to 0.20, slight agreement; from 0.20 to 0.40, fair agreement; from 0.40 to 0.60, moderate agreement; from 0.60 to 0.80, substantial agreement; and from 0.80 to 1.00, almost perfect agreement^(49)^.

Last, the AC1 test was used with two or more listeners and with a rating scale with two or more categories. In this study, AC1 test was used only for intrarater reliability in the perceptual-auditory judgment of OS using a numerical scale GRBAS, and predominant vocal deviation. AC1 ranges from 0 to 1 and is interpreted as follows: the closer to 1 AC1 is, the better the agreement is (the less likely interrater agreement will be random)^(50)^. The Kruskall-Wallis test and the Nemeny test were used to verify whether there was a difference in the mean intrarater reliability values of among the speech tasks investigated.

All statistical tests were done in the free software environment R (v3.3.1). A 5% significance level was chosen for all tests.

## RESULTS

The results section was divided into two parts, according to the type of scale used in the auditory-perceptual judgment: VAS and GRBAS.

### Results of intra and interrater reliability in the auditory-perceptual judgment of os with using VAS

The overall mean for the groups in the sustained vowel /ɛ/ task was 0.63. Group 1, composed of undergraduate students in Speech-Language Pathology without experience (GS-U), showed a mean of 0.5818. Group 2, consisting of undergraduate students with experience (GS-T), demonstrated a mean of 0.6909. The third group, comprising speech-language pathologists non-specialized in voice (SLP-NV), presented a mean of 0.7137, while the fourth group, composed of voice-specialized speech-language pathologists (SLP-V), displayed a mean of 0.5777. Regarding the sustained vowel /a/ task, the overall mean for the groups was 0.64. Group 1 (GS-U) had a mean of 0.5818. Group 2 (GS-T) exhibited a mean of 0.6392. The third group (SLP-NV) showed a mean of 0.8099, while the fourth group (SLP-V) displayed a mean of 0.6193.

For the speech sentences task, the overall mean for the groups was 0.81. Group 1 (GS-U) had a mean of 0.8013. Group 2 (GS-T) demonstrated a mean of 0.8292. The third group (SLP-NV) presented a mean of 0.8264, while the fourth group (SLP-V) of pathologists showed a mean of 0.8187. As for the counting task, the overall mean for the groups was 0.73. Group 1 (GS-U) had a mean of 0.6838. Group 2 (GS-T) showed a mean of 0.7466. The third group (SLP-NV) presented a mean of 0.7926, while the fourth group (SLP-V) displayed a mean of 0.7926.

**Figure 2:**
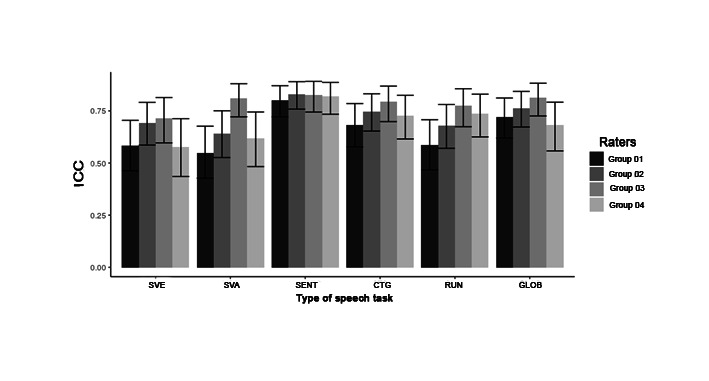
Mean ICC for interrater reliability in the auditory-perceptual analysis of overall severity using the VAS

The overall mean for the groups in the running speech task was 0.68. Group 1 (GS-U) showed a mean of 0.5856. Group 2 (GS-T) exhibited a mean of 0.6775. The third group (SLP-NV) presented a mean of 0.7741, while the fourth group (SLP-V) displayed a mean of 0.7362. Lastly, the overall mean for the groups in the interconnected tasks was 0.74. Group 1 (GS-U) had a mean of 0.719. Group 2 (GS-T) demonstrated a mean of 0.7625. The third group (SLP-NV) presented a mean of 0.8128, while the fourth group (SLP-V) showed a mean of 0.6825. Furthermore, Figure 2 complements the data on the mean ICC for interrater reliability in the auditory-perceptual analysis of overall severity using the VAS.

**Caption:** SVE (sustained vowel /ɛ/); SVA (sustained vowel /a/); SENT (sentences); CTG (counting); RUN (running speech); GLOB (interconnected tasks). Group 1 (undergraduate students in Speech-Language Pathology without experience); Group 2 (undergraduate students with experience); Group 3 (general speech-language pathologists); Group 4 (voice specialized speech-language pathologists)

Sentences obtained the best ICC among the listener groups, with good interrater reliability in each group, using the VAS. In general, the sustained vowel /ɛ/ obtained the highest ICC interval amplitudes in all groups, indicating lower interrater reliability in their respective groups.

In general, the SLP-NV group demonstrated the best interrater reliability in OS judgment in different speech tasks, obtaining good reliability in all investigated tasks. On the other hand, the GS-U group presented the lowest ICC values in the auditory-perceptual judgment of OS in all speech tasks studied.

As for intrarater reliability (Table 1), in general, the SLP-V group showed the highest agreement in the auditory-perceptual judgment of OS in all speech tasks investigated. There was a statistically significant difference between the groups regarding intrarater reliability in the auditory-perceptual judgment of OS in the vowel /a/. The SLP-NV and SLP-V groups showed greater intrarater reliability in evaluating OS with the vowel /a/. The listeners of all groups showed good reliability (0.75 |-0.90) with the tasks with continued speech (sentences, number counting, and running speech).

**Table 1:** Intrarater reliability in the auditory-perceptual judgment using a visual analog scale

Task type	Participant	Group	ICC	Inferior limit	Upper limit	p-value
SVE (total mean = 0.63)	1	SLP-V	0.8465	0.2391	0.9824	0.00887
GS-U 0.4835	2	SLP-NV	0.8778	0.3508	0.9862	0.00514
GS-T 0.5280	3	SLP-NV	0.8508	0.2536	0.9829	0.00829
SLP-NV 0.7494	4	SLP-V	0.9021	0.4492	0.9891	0.00301
SLP-V 0.8160	5	SLP-V	0.7413	0	0.9688	0.03017
p-value: 0.09186	6	SLP-NV	0.486	0	0.9288	0.13745
	7	SLP-NV	0.7828	0.05265	0.9743	0.02012
	8	SLP-V	0.7741	0.03044	0.9732	0.02206
	9	GS-T	0.8394	0.2159	0.9815	0.00988
	10	GS-T	0.3116	0	0.8939	0.24789
	11	GS-T	0.6474	0	0.9553	0.06085
	12	GS-U	0	0	0.01561	0.97359
	13	GS-U	0.7322	0	0.9675	0.03267
	14	GS-U	0	0	0.807	0.48566
	15	GS-U	0.5739	0	0.9438	0.09219
	16	GS-U	0.7681	0.01575	0.9724	0.02344
	17	GS-U	0.7317	0	0.9675	0.0328
	18	GS-U	0.5786	0	0.9446	0.09001
	19	GS-T	0.6961	0	0.9625	0.04357
	20	GS-T	0	0	0.7535	0.59349
	21	GS-T	0.9037	0.456	0.9893	0.00289
	22	GS-T	0.298	0	0.8908	0.25742
SVA (total mean = 0.70)	1	SLP-V	0.826	0.1736	0.9798	0.01196
GS-U 0.6295	2	SLP-NV	0.8599	0.2851	0.984	0.00713
GS-T 0.4289	3	SLP-NV	0.9332	0.5933	0.9926	0.00118
SLP-NV 0.8394	4	SLP-V	0.8798	0.3582	0.9864	0.00494
SLP-V 0.8677	5	SLP-V	0.772	0.02531	0.9729	0.02254
p-value: 0.01898	6	SLP-NV	0.7533	0	0.9704	0.02705
	7	SLP-NV	0.811	0.1293	0.978	0.01453
	8	SLP-V	0.9932	0.9511	0.9993	< 0.0005
	9	GS-T	0.6162	0	0.9505	0.0734
	10	GS-T	0.6287	0	0.9525	0.06824
	11	GS-T	0.741	0	0.9687	0.03025
	12	GS-U	0.4976	0	0.9308	0.131
	13	GS-U	0.76	0	0.9713	0.02538
	14	GS-U	0.9412	0.6344	0.9936	0.00087
	15	GS-U	0.5832	0	0.9453	0.08788
	16	GS-U	0.454	0	0.9229	0.15579
	17	GS-U	0.9154	0.5078	0.9906	0.00211
	18	GS-U	0.2553	0	0.8809	0.28812
	19	GS-T	0.0723	0	0.8309	0.42826
	20	GS-T	0.7477	0	0.9696	0.02848
	21	GS-T	0.0065	0	0.8093	0.48048
	22	GS-T	0.1897	0	0.8644	0.33706
SENTENCES (total mean = 0.80)	1	SLP-V	0.9633	0.7575	0.996	< 0.0005
GS-U 0.7789	2	SLP-NV	0.8944	0.4165	0.9882	0.00362
GS-T 0.8170	3	SLP-NV	0.6515	0	0.9559	0.05926
SLP-NV 0.8089	4	SLP-V	0.9457	0.6584	0.9941	0.00071
SLP-V 0.8232	5	SLP-V	0.9447	0.653	0.994	0.00074
p-value: 0.47080	6	SLP-NV	0.7732	0.02821	0.973	0.02227
	7	SLP-NV	0.9164	0.5124	0.9907	0.00205
	8	SLP-V	0.439	0	0.9201	0.16477
	9	GS-T	0.7241	0	0.9664	0.03497
	10	GS-T	0.7875	0.06472	0.9749	0.01913
	11	GS-T	0.9078	0.4737	0.9897	0.0026
	12	GS-U	0.9137	0.5004	0.9904	0.00221
	13	GS-U	0.5085	0	0.9327	0.12512
	14	GS-U	0.8576	0.2768	0.9838	0.00742
	15	GS-U	0.8414	0.2224	0.9818	0.00959
	16	GS-U	0.9034	0.4548	0.9892	0.00291
	17	GS-U	0.9149	0.5057	0.9906	0.00214
	18	GS-U	0.5127	0	0.9335	0.12283
	19	GS-T	0.9168	0.5142	0.9908	0.00203
	20	GS-T	0.5928	0	0.9469	0.08353
	21	GS-T	0.9341	0.5977	0.9927	0.00115
	22	GS-T	0.8557	0.2701	0.9835	0.00766
CTG (total mean = 0.83)	1	SLP-V	0.9504	0.6836	0.9946	0.00057
GS-U 0.8532	2	SLP-NV	0.8618	0.2916	0.9843	0.00691
GS-T 0.7897	3	SLP-NV	0.935	0.6022	0.9928	0.00111
SLP-NV 0.8294	4	SLP-V	0.9045	0.4592	0.9893	0.00284
SLP-V 0.8973	5	SLP-V	0.7547	0	0.9706	0.0267
p-value: 0.46550	6	SLP-NV	0.6315	0	0.9529	0.06711
	7	SLP-NV	0.8893	0.3956	0.9876	0.00405
	8	SLP-V	0.9798	0.8596	0.9978	< 0.0005
	9	GS-T	0	0	0.807	0.48566
	10	GS-T	0.8815	0.3648	0.9866	0.00478
	11	GS-T	0.9476	0.6685	0.9943	0.00065
	12	GS-U	0.8667	0.3092	0.9849	0.00634
	13	GS-U	0.7644	0.00681	0.9719	0.02431
	14	GS-U	0.8271	0.177	0.98	0.01178
	15	GS-U	0.8222	0.1622	0.9794	0.01258
	16	GS-U	0.8456	0.2362	0.9823	0.00899
	17	GS-U	0.9936	0.9535	0.9993	< 0.0005
	18	GS-U	0.8527	0.2598	0.9832	0.00805
	19	GS-T	0.9608	0.7423	0.9957	< 0.0005
	20	GS-T	0.8846	0.3769	0.987	0.00448
	21	GS-T	0.9759	0.8347	0.9974	< 0.0005
	22	GS-T	0.8775	0.3495	0.9862	0.00517
RUN (total mean = 0.86)	1	SLP-V	0.8653	0.3041	0.9847	0.0065
GS-U 0.8938	2	SLP-NV	0.9149	0.5056	0.9906	0.00214
GS-T 0.8127	3	SLP-NV	0.9703	0.7998	0.9968	< 0.0005
SLP-NV 0.8850	4	SLP-V	0.942	0.6386	0.9936	0.00084
SLP-V 0.9281	5	SLP-V	0.9332	0.5931	0.9926	0.00119
p-value: 0.96690	6	SLP-NV	0.9891	0.9222	0.9988	< 0.0005
	7	SLP-NV	0.6659	0	0.9581	0.05396
	8	SLP-V	0.9719	0.8097	0.997	< 0.0005
	9	GS-T	0.952	0.6927	0.9948	0.00053
	10	GS-T	0.9328	0.5914	0.9926	0.0012
	11	GS-T	0.9507	0.6852	0.9946	0.00056
	12	GS-U	0.9596	0.7356	0.9956	< 0.0005
	13	GS-U	0.9027	0.4515	0.9891	0.00297
	14	GS-U	0.9773	0.8435	0.9975	< 0.0005
	15	GS-U	0.9848	0.8931	0.9984	< 0.0005
	16	GS-U	0.5793	0	0.9447	0.08968
	17	GS-U	0.8811	0.3633	0.9866	0.00481
	18	GS-U	0.972	0.8099	0.997	< 0.0005
	19	GS-T	0.9291	0.5731	0.9922	0.00137
	20	GS-T	0	0	0.759	0.58371
	21	GS-T	0.9434	0.6461	0.9938	0.00079
	22	GS-T	0.981	0.8676	0.998	< 0.0005

**Caption:** SVE (sustained vowel /ɛ/); SVA (sustained vowel /a/); SENT (sentences); CTG (counting); RUN (running speech). GS-U (undergraduate students in Speech-Language Pathology without experience); GS-T (undergraduate students with experience); SLP-NV (speech-language pathologists non specialized in voice); SLP-V (voice specialized speech-language pathologists); ICC (intraclass correlation coefficient)

### Results of intra and interrater reliability in the auditory-perceptual judgment of os with using GRBAS

**Figure 3:**
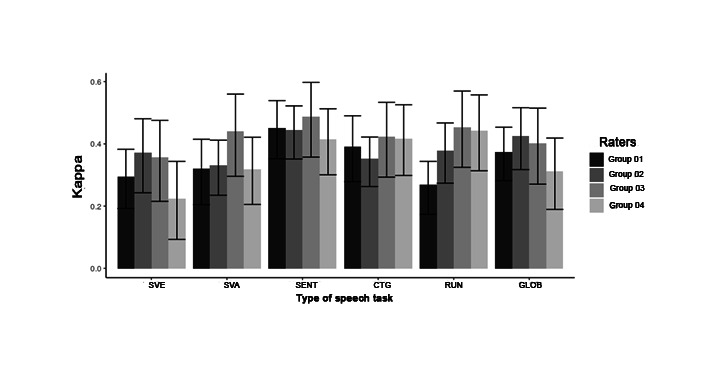
Mean Kappa for interrater reliability in the auditory-perceptual analysis of overall severity using the GRBAS

Table 2 and Figure 3 present the mean Kappa results for interrater reliability in the auditory-perceptual analysis of voice quality using the GRBAS. Sentences obtained the best ICC among the listener groups, with moderate interrater reliability in each group, using the GRBAS. In general, the sustained vowel /ɛ/ showed the lowest interrater reliability in all groups.

**Table 2:** Interrater reliability by group in the auditory-perceptual analysis using GRBAS

**Task type**	**Group**	**Mean kappa**
SVE	GS-U	0.2954
Overall group mean:		
	GS-T	0.3716
(0.30)		
	SLP-NV	0.3576
	SLP-V	0.2233
SVA	GS-U	0.3205
Overall group mean:	GS-T	0.3319
(0.35)	SLP-NV	0.4409
	SLP-V	0.3176
SENT	GS-U	0.4511
Overall group mean:		
	GS-T	0.4447
(0.44)		
	SLP-NV	0.4866
	SLP-V	0.4149
CTG	GS-U	0.3911
Overall group mean:	GS-T	0.3519
(0.39)		
	SLP-NV	0.4232
	SLP-V	0.4158
RUN	GS-U	0.2699
Overall group mean:		
(0.38)	GS-T	0.3772
	SLP-NV	0.4529
	SLP-V	0.4415
GLOB	GS-U	0.3747
Overall group mean:	GS-T	0.4257
(0.37)		
	SLP-NV	0.402
	SLP-V	0.311

**Caption**: SVE (sustained vowel /ɛ/); SVA (sustained vowel /a/); SENT (sentences); CTG (counting); RUN (running speech); GLOB (interconnected tasks). GS-U (undergraduate students in Speech-Language Pathology without experience); GS-T (undergraduate students with experience); SLP-NV (speech-language pathologists non specialized in voice); SLP-V (voice specialized speech-language pathologists)

**Caption:** SVE (sustained vowel /ɛ/); SVA (sustained vowel /a/); SENT (sentences); CTG (counting); RUN (running speech); GLOB (interconnected tasks). Group 1 (undergraduate students in Speech-Language Pathology without experience); Group 2 (undergraduate students with experience); Group 3 (general speech-language pathologists); Group 4 (voice specialized speech-language pathologists)

SLP-NV group presented better interrater reliability in the auditory-perceptual judgment of the “G” in the GRBAS numerical scale for all studied speech tasks.SLP-V group demonstrated the highest intrarater reliability (Table 3) in all speech tasks using the GRBAS scale. The running speech task presented higher intrarater reliability in the studied groups when they used GRBAS.

**Table 3:** Intrarater reliability in the perceptual-auditory judgment of overall severity using a GBRASI

**Task type**	**Participants**	**Group**	** **	**AC1**	**Inferior limit.**	**Upper limit**
**SVE** (Mean=0.6003)	1	SLP-V		0.7744	0.1553	1
**GS-U** 0.5782	2	SLP-NV		1	1	1
**GS-T** 0.3728	3	SLP-NV		0.5041	0	1
**SLP-NV** 0.6848	4	SLP-V		1	1	1
**SLP-V** 0.7655	5	SLP-V		0.76	0.164	1
	6	SLP-NV		0.4872	0	1
	7	SLP-NV		0.7479	0.1237	1
	8	SLP-V		0.5276	0	1
	9	GS-T		0.5082	0	1
	10	GS-T		0.0083	0	0.6694
	11	GS-T		0.4915	0	1
	12	GS-U		0.7674	0.1799	1
	13	GS-U		1	1	1
	14	GS-U		0.2623	0	1
	15	GS-U		0.5238	0	1
	16	GS-U		0.5122	0	1
	17	GS-U		0.4737	0	1
	18	GS-U		0.5082	0	1
	19	GS-T		0.5238	0	1
	20	GS-T		0.3023	0	1
	21	GS-T		0.0083	0	0.6694
	22	GS-T		0.7674	0.1799	1
**SVA** (Mean: 0.4505)	1	SLP-V		0.2035	0	1
**GS-U** 0.2140	2	SLP-NV		0.7521	0.121	1
**GS-T** 0.4041	3	SLP-NV		0.76	0.164	1
**SLP-NV** 0.5050	4	SLP-V		1	1	1
**SLP-V** 0.6789	5	SLP-V		0.5122	0	1
	6	SLP-NV		0	0	0.6376
	7	SLP-NV		0.5082	0	1
	8	SLP-V		1	1	1
	9	GS-T		0.2308	0	1
	10	GS-T		0.2913	0	1
	11	GS-T		0	0	0.5893
	12	GS-U		0.0164	0	0.7071
	13	GS-U		0.4783	0	1
	14	GS-U		0	0	0.6715
	15	GS-U		0.2562	0	1
	16	GS-U		0.2562	0	1
	17	GS-U		0.4915	0	1
	18	GS-U		0	0	0.6656
	19	GS-T		0.2308	0	1
	20	GS-T		1	1	1
	21	GS-T		0.5522	0	1
	22	GS-T		0.5238	0	1
**RUN** (Mean: 0.7511)	1	SLP-V		1	1	1
**GS-U** 0.6513	2	SLP-NV		1	1	1
**GS-T** 0.7265	3	SLP-NV		0.7436	0.1004	1
**SLP-NV** 0.8728	4	SLP-V		1	1	1
**SLP-V** 0.7540	5	SLP-V		0.7479	0.1237	1
	6	SLP-NV		1	1	1
	7	SLP-NV		0.7479	0.1237	1
	8	SLP-V		0.2683	0	1
	9	GS-T		0.7638	0.1593	1
	10	GS-T		0.7744	0.1553	1
	11	GS-T		0.7674	0.1799	1
	12	GS-U		1	1	1
	13	GS-U		0.2913	0	1
	14	GS-U		0.5082	0	1
	15	GS-U		0.5082	0	1
	16	GS-U		1	1	1
	17	GS-U		0.7479	0.1237	1
	18	GS-U		0.5041	0	1
	19	GS-T		0.7479	0.1237	1
	20	GS-T		0.5238	0	1
	21	GS-T		1	1	1
	22	GS-T		0.5082	0	1
**SENT** (Mean: 0,6709)	1	SLP-V		0.7561	0.1178	1
**GS-U** 0.5614	2	SLP-NV		0.4915	0	1
**GS-T** 0.6341	3	SLP-NV		1	1	1
**SLP-NV** 0.6108	4	SLP-V		1	1	1
**SLP-V** 0.8775	5	SLP-V		0.76	0.164	1
	6	SLP-NV		0.2174	0	1
	7	SLP-NV		0.7345	0.0809	1
	8	SLP-V		1	1	1
	9	GS-T		1	1	1
	10	GS-T		0.2308	0	1
	11	GS-T		0.7561	0.1178	1
	12	GS-U		0.5041	0	1
	13	GS-U		1	1	1
	14	GS-U		0.4958	0	1
	15	GS-U		0.2174	0	1
	16	GS-U		0.7436	0.1013	1
	17	GS-U		0.7521	0.121	1
	18	GS-U		0.2174	0	1
	19	GS-T		0.4737	0	1
	20	GS-T		1	1	1
	21	GS-T		0.2308	0	1
	22	GS-T		0.7479	0.1237	1
**CTG** (Mean: 0.7437)	1	SLP-V		0.7479	0.1237	1
**GS-U** 0.7119	2	SLP-NV		0.4915	0	1
**GS-T** 0.8906	3	SLP-NV		0.76	0.164	1
**SLP-NV** 0.5628	4	SLP-V		0.7436	0.1013	1
**SLP-V** 0.8098	5	SLP-V		1	1	1
	6	SLP-NV		1	1	1
	7	SLP-NV		0	0	0.7002
	8	SLP-V		0.7479	0.1237	1
	9	GS-T		1	1	1
	10	GS-T		1	1	1
	11	GS-T		1	1	1
	12	GS-U		0.2437	0	1
	13	GS-U		0.7436	0.1004	1
	14	GS-U		1	1	1
	15	GS-U		0.7436	0.1013	1
	16	GS-U		0.7561	0.1178	1
	17	GS-U		0.7479	0.1237	1
	18	GS-U		0.7521	0.121	1
	19	GS-T		0.7391	0.0769	1
	20	GS-T		0.7521	0.121	1
	21	GS-T		0.7436	0.1013	1
	22	GS-T		1	1	1

**Caption**: SVE (sustained vowel /ɛ/); SVA (sustained vowel /a/); SENT (sentences); CTG (counting); RUN (running speech). GS-U (undergraduate students in Speech-Language Pathology without experience); GS-T (undergraduate students with experience); SLP-NV (speech-language pathologists non specialized in voice); SLP-V (voice specialized speech-language pathologists), AC1 (agreement coefficient).

In general, GRBAS had lower reliability values than the VAS.

### Results of intra and interrater reliability in the auditory-perceptual judgment of predominant vocal deviation

**Figure 4:**
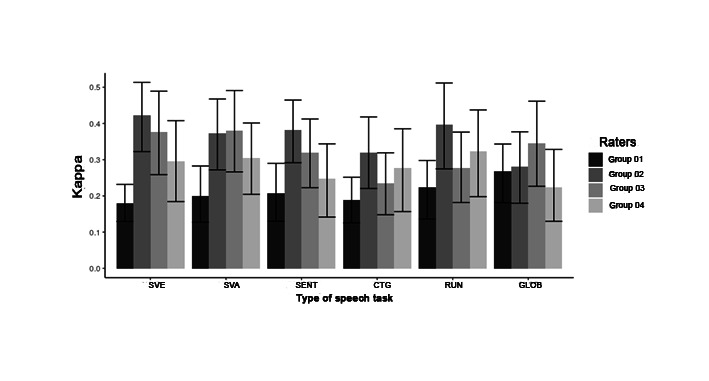
Mean kappa for interrater reliability in the auditory-perceptual analysis of predominant voice quality

Table 4 and Figure 4 present the mean Kappa results for interrater reliability in the auditory-perceptual analysis of predominant voice quality. Sustained vowel (/a/ and /ɛ/) and running speech shows the best interrater reliability between groups of judges. GS-T group obtained the best result of interrater reliability in judging the predominant vocal deviation for most speech tasks (sustained vowel /ɛ/, sentences, counting, and running speech). The GS-T group showed regular agreement (0.20 |- 0.40) in all these tasks, except for the sustained vowel /ɛ/, where a moderate agreement was found (0.40 |- 0.60). On the other hand, the SLP-NV group obtained better interrater reliability to listener the predominant vocal deviation related to the vowel /a/ and for the five speech tasks together. In these two tasks, the SLP-NV group listeners showed regular agreement (0.20 |- 0.40).

**Table 4:** Interrater reliability by group in the auditory-perceptual analysis of predominant voice quality

**Task type**	**Group**	**kappa**
SVE	GS-U	0.1803
Overall group mean:		
(0.31)	GS-T	0.4229
	SLP-NV	0.3756
	SLP-V	0.2947
SVA	GS-U	0.2006
Overall group mean:		
(0.31)	GS-T	0.3731
	SLP-NV	0.3794
	SLP-V	0.3051
SENT	GS-U	0.2078
Overall group mean:	GS-T	0.3819
(0.28)	SLP-NV	0.32
	SLP-V	0.247
CTG	GS-U	0.1885
Overall group mean:		
(0.24)		
	GS-T	0.319
	SLP-NV	0.2349
	SLP-V	0.2772
RUN	GS-U	0.2229
Overall group mean:		
(0.30)	GS-T	0.3969
	SLP-NV	0.2776
	SLP-V	0.3235
GLOB	GS-U	0.267
Overall group mean:		
(0.27)	GS-T	0.2805
	SLP-NV	0.3455
	SLP-V	0.2238

**Caption**: SVE (sustained vowel /ɛ/); SVA (sustained vowel /a/); SENT (sentences); CTG (counting); RUN (running speech); GLOB (interconnected tasks). GS-U (undergraduate students in Speech-Language Pathology without experience); GS-T (undergraduate students with experience); SLP-NV (speech-language pathologists non specialized in voice); SLP-V (voice specialized speech-language pathologists).

As for intrarater reliability in the assessment of predominant vocal deviation, SLP-NV presented higher GAC1 values ​​for the tasks of the sustained vowel /ɛ/, running speech, and sentences (Table 5). In addition, the SLP-V group showed higher values ​​in GAC1 in tasks related to sustained vowel /a/ and counting. In general, there was a higher intrarater reliability in the judgment of the predominant vocal quality for the running speech and counting tasks.

**Table 5:** Intrarater reliability in the perceptual-auditory judgment of predominant vocal deviation

**Task type**	**Participants**	**Group**		**AC1**	**Inferior limit.**	**Upper limit**
SVE (Mean = 0.5708)	1	SLP-V		0.7391	0.0769	1
GS-U = 0.5328	2	SLP-NV		0.7436	0.1013	1
GS-T =0.5707	3	SLP-NV		0.4915	0	1
SLP-NV = 0.6207	4	SLP-V		0.4872	0	1
SLP-V = 0.5591	5	SLP-V		0.2623	0	1
	6	SLP-NV		0.7521	0.121	1
	7	SLP-NV		0.4958	0	1
	8	SLP-V		0.7479	0.1237	1
	9	GS-T		0.4958	0	1
	10	GS-T		0.4915	0	1
	11	GS-T		0	0	0.6715
	12	GS-U		0.7521	0.121	1
	13	GS-U		1	1	1
	14	GS-U		0.2913	0	1
	15	GS-U		0.2035	0	1
	16	GS-U		0.4915	0	1
	17	GS-U		0.7479	0.1237	1
	18	GS-U		0.2437	0	1
	19	GS-T		0.7479	0.1237	1
	20	GS-T		0.7479	0.1237	1
	21	GS-T		0.7561	0.1178	1
	22	GS-T		0.7561	0.1178	1
SVA (Mean = 0.3719)	1	SLP-V		0.76	0.164	1
GS-U = 0.2927	2	SLP-NV		0.5238	0	1
GS-T =0.2881	3	SLP-NV		0	0	0.2548
SLP-NV = 0.3983	4	SLP-V		0.4783	0	1
SLP-V = 0.5085	5	SLP-V		0.5522	0	1
	6	SLP-NV		0.7674	0.1799	1
	7	SLP-NV		0.3023	0	1
	8	SLP-V		0.2437	0	1
	9	GS-T		0.2373	0	1
	10	GS-T		0.4872	0	1
	11	GS-T		0	0	0.2585
	12	GS-U		0	0	0.1821
	13	GS-U		0.4958	0	1
	14	GS-U		0.0244	0	0.7001
	15	GS-U		0.2308	0	1
	16	GS-U		0.5041	0	1
	17	GS-U		0.5082	0	1
	18	GS-U		0.2857	0	1
	19	GS-T		0.5122	0	1
	20	GS-T		0.5238	0	1
	21	GS-T		0	0	0.6832
	22	GS-T		0.2562	0	1
RUN (Mean = 0.7321)	1	SLP-V		0.7872	0.2289	1
GS-U = 0.6891	2	SLP-NV		1	1	1
GS-T =0.7477	3	SLP-NV		0.5489	0	1
SLP-NV = 0.8340	4	SLP-V		1	1	1
SLP-V = 0.6576	5	SLP-V		0.5522	0	1
	6	SLP-NV		1	1	1
	7	SLP-NV		0.7872	0.2289	1
	8	SLP-V		0.2913	0	1
	9	GS-T		0.5238	0	1
	10	GS-T		0.7744	0.1553	1
	11	GS-T		0.7872	0.2289	1
	12	GS-U		0.5041	0	1
	13	GS-U		1	1	1
	14	GS-U		1	1	1
	15	GS-U		0	0	0.5893
	16	GS-U		1	1	1
	17	GS-U		0.7674	0.1799	1
	18	GS-U		0.5522	0	1
	19	GS-T		0.7872	0.2289	1
	20	GS-T		0.7872	0.2289	1
	21	GS-T		0.7872	0.2289	1
	22	GS-T		0.7872	0.2289	1
SENT (Mean = 0.5691)	1	SLP-V		0.7521	0.0945	1
GS-U = 0.4774	2	SLP-NV		0.7561	0.1178	1
GS-T =0.6957	3	SLP-NV		1	1	1
SLP-NV = 0.3983	4	SLP-V		0.5489	0	1
SLP-V = 0.7051	5	SLP-V		0.7521	0.121	1
	6	SLP-NV		0.5122	0	1
	7	SLP-NV		0.4915	0	1
	8	SLP-V		0.7674	0.1799	1
	9	GS-T		0.5276	0	1
	10	GS-T		1	1	1
	11	GS-T		0.5238	0	1
	12	GS-U		0.2308	0	1
	13	GS-U		0.7674	0.1799	1
	14	GS-U		0.7744	0.1553	1
	15	GS-U		0	0	0.7006
	16	GS-U		0.5122	0	1
	17	GS-U		0.5489	0	1
	18	GS-U		0.5082	0	1
	19	GS-T		0.7479	0.1237	1
	20	GS-T		0.5276	0	1
	21	GS-T		0.7872	0.2289	1
	22	GS-T		0.7561	0.1178	1
CTG (Mean = 0.7108)	1	SLP-V		0.4872	0	1
GS-U = 0.7534	2	SLP-NV		0.7674	0.1799	1
GS-T =0.6891	3	SLP-NV		0.4872	0	1
SLP-NV = 0.7572	4	SLP-V		0.7521	0.0945	1
SLP-V = 0.6438	5	SLP-V		0.5489	0	1
	6	SLP-NV		0.7744	0.1553	1
	7	SLP-NV		1	1	1
	8	SLP-V		0.7872	0.2289	1
	9	GS-T		0.5082	0	1
	10	GS-T		0.7638	0.1593	1
	11	GS-T		0.7521	0.0945	1
	12	GS-U		0.4915	0	1
	13	GS-U		0.7674	0.1799	1
	14	GS-U		0.7744	0.1553	1
	15	GS-U		1	1	1
	16	GS-U		0.4737	0	1
	17	GS-U		1	1	1
	18	GS-U		0.7674	0.1799	1
	19	GS-T		0.5122	0	1
	20	GS-T		0.5238	0	1
	21	GS-T		1	1	1
	22	GS-T		0.7638	0.1593	1

**Legend**: SVE (sustained vowel /ɛ/); SVA (sustained vowel /a/); SENT (sentences); CTG (counting); RUN (running speech); GS-U (undergraduate students in Speech-Language Pathology without experience); GS-T (undergraduate students with experience); SLP-NV (speech-language pathologists non specialized in voice); SLP-V (voice specialized speech-language pathologists); AC1 (agreement coefficient)

## DISCUSSION

Several factors are known to affect the auditory-perceptual evaluation of voice quality, including listener experience, speech task, and measurement scale used^(16,24,51)^. The roles of these factors in the auditory-perceptual analysis of voice are not entirely clear, and they must be investigated to assess their influence on the reliability of voice analysis^(12,16)^.

In the present study, we observed that the listener experience influenced the intra and interrater reliability in the auditory-perceptual evaluation of voice quality in both the VAS and GRBAS scales. In our study, we adopted a perspective in which listener experience basically has two domains: the quantitative and the qualitative domains^(24,45,52)^. The quantitative domain refers to the experience time of this subject, for example, the number of years for which a specific listener has been conducting auditory-perceptual evaluation of voice. Conversely, the qualitative domain refers to the type of experience of the individual, for example, the predominant profile of patients this SLP had treated throughout his or her career (behavioral dysphonia, neurogenic disorders, for example), or even the type of training in auditory-perceptual evaluation of voice that this healthcare professional had completed^(11,52)^.

As expected, untrained listeners (GS-U) demonstrated low intra and interrater reliability in the auditory-perceptual analysis of voice quality using the VAS and GRBAS scales. Other studies have found low interrater reliability rate among untrained listeners^(1)^. Thus, these individuals likely lacked internal standards robust enough to reliably listener the parameters listed in this study. In general, listeners with no experience in auditory-perceptual evaluation have much broader internal standards for NVVQ because they have heard “healthy” voices throughout their lives much more frequently than voices with some form of deviation^(1,16,22,24)^. Thus, apparently, the strategy used by this group (untrained students) would precisely rely on the comparison with these internal standards; hence, stimuli noticeably more similar to the internal pattern of “healthy” voices were more easily associated with the NVVQ parameter, whereas voices farther from this pattern were more commonly associated with severe vocal deviation. Therefore, the number of voices rated with mild and moderate vocal deviations was lower in this group than in the other groups, and this rating range was one of the most susceptible to variation^(1,53)^.

We expected that the group with the most extended experience in auditory-perceptual judgment would obtain the best values of inter and intrarater reliability^(12,54)^. In general, experience is expected to separately affect interrater reliability positively^(55)^. However, in our study, experience time was not associated with increased interrater reliability, but it seems that experience with the deliberate practice of auditory-perceptual training seems to have an additional influence on the judges' reliability. training time and the type of training received by the judges.

In general, we observed that the SLP group that underwent the same type of auditory-perceptual training (SLP-NV) demonstrated greater interrater reliability in the auditory-perceptual judgment, using VAS and GRBAS as well as in the assessment of predominant vocal quality. In this way, it appears that participation in the same standardized training improves interrater reliability. Standardized training likely allows participants to develop similar internal reference standards, which improves agreement among listeners. The GS-T obtained the second best interrater reliability can confirm that the standardized training seems to improve the agreement between listeners. These two groups were made up of subjects with the same training in auditory-perceptual evaluation of voice quality.

The results of this study regarding the groups of undergraduate speech-language pathology students (Group 2) and general speech-language pathologists (Group 3) indicate that this type of training was the main factor affecting interrater reliability in the auditory-perceptual analysis of voice. Several studies also highlight the importance of training for increasing interrater reliability in the auditory-perceptual evaluation of voice quality^(1,15,53,56)^.

In turn, experience time seems to be a more determining factor in intrarater reliability. In general, SLP-V demonstrated greater intrarater reliability in the auditory-perceptual judgment of vocal quality using both the VAS and GRBAS scales. On the other hand, SLP-NV only demonstrates greater intra reliability for the judgment of predominant voice quality.

Although the SLP-V group in this study consists of SLP with about the same experience time in the auditory-perceptual evaluation of voice quality (more than ten years), the type of experience of these subjects varied considerably. Therefore, this was the most heterogeneous group regarding experience quality/type. Each of the four members of the group worked in different areas of speech-language pathology, from monitoring patients with head and neck cancer to enhancing the professional voice users, undergoing different types of training throughout their careers.

Thus, the internal parameters of the experienced subjects were likely shaped according to their type of experience and training over the years. A clinician whose professional experience is most commonly related to neurological disorders likely has different internal reference standards from another speech-language pathologist more experienced in enhancing the voice of singers, for example^(11,57)^. In addition, a professional whose training was more focused on phonetic evaluation of vocal quality may not have the same standards as a clinician whose training primarily focused on the VAS, and vice versa^(11,16,22,57)^. All these factors may be possible sources of variability among the listeners.

All groups of investigated listeners presented lower interrater reliability values for the judgment of predominant vocal quality compared to OS's judgment in VAS and GRBAS. The low reliability values of the voice quality parameter may be explained by a few reasons. The definition of voice quality is quite complex, involving various parameters and nomenclatures^(16,55)^. In addition, identifying a single predominant feature in a natural voice segment, that is, originating from a human phonatory system, is apparently more difficult^(1,53)^.

As for the speech tasks, we observed that the use of continuous speech tasks obtained the best values of inter (sentences) and intrarater (count, sentences, and running speech) reliability in both VAS and GRBAS scales, as well as the highest intrarater reliability in judging the predominant vocal quality. On the other hand, the sustained vowel tasks only produced greater interrater reliability in the judgment of the predominant vocal quality.

On the effect of speech task on the auditory-perceptual evaluation of voice quality, the hypothesis of our study was confirmed: auditory-perceptual analysis was affected by the speech task. In general, no significant difference was found in interrater reliability for the tasks sustained vowel /ɛ/ and sustained vowel /a/, with an overall mean of 0.63 and 0.64, respectively, using the VAS. According to the ICC, thus representing moderate reliability (0.50 < ICC < 0.75). For the GRBAS, the values were lower, with an overall mean of 0.30 for sustained vowel /ɛ/, and 0.35 for sustained vowel /a/, both corresponding to fair interrater reliability, according to the kappa (0.20 < kappa < 0.40). GS-T and SLP-NV had a slightly higher interrater reliability coefficient for sustained vowel /ɛ/ and sustained vowel /a/.

We observed that there was a statistically significant difference between the groups regarding intrarater reliability in the auditory-perceptual judgment of OS in the vowel /a/. Specifically. The GS-T showed a lower reliability compared to the other groups, although it is still considered a moderate reliability. In a speculative way, we sought to understand what would justify this difference in the GS-T in relation to the other groups. The GS-T was the group with the shortest temporal distance between the deliberate practice of auditory-perceptual training and data collection in this research. The structure of the training carried out in the undergraduate course uses the vowel /ɛ/ associated with other linked speech tasks. Thus, considering that the process of learning perceptual tasks works with memory access, the recent training they underwent with a single vocal (/ɛ/), may have influenced the reliability result for the vowel /a/.

Connected speech tasks, in general, had higher mean interrater reliability values within the groups in the perceptual evaluation of voice quality than the sustained vowel tasks. The task sentences had the best reliability in each group of raters, with substantial agreement (0.75 < ICC < 0.90) and an overall mean among the groups of 0.81 for the VAS and with moderate agreement (0.40 < kappa < 0.60) and an overall mean of 0.44 for GRBAS. Once again, the GS-T and SLP-NV group had slightly higher reliability values for this task than the other groups.

Although sentences was the task with the highest reliability with both the VAS and GRBAS, in the analysis of the predominant voice quality, the highest values were found in the sustained vowel tasks. This could be because the sustained vowel task involves a more stable configuration of the larynx and vocal tract than connected speech, which facilitates the identification and analysis of specific parameters, such as the presence or absence of roughness^(13,19,25)^.

In our study, CAPE-V sentences had the best agreement among all tasks in all study groups. These results reinforce how reliable the data from this protocol are and that they have good reproducibility for scientific research^(38,58,59)^.

Among all speech tasks used in our study, sentences alone (without being combined with other tasks, such as the stimulus “interconnected tasks”) was the longest, ranging from 12 to 14 seconds. A longer speech task provides a higher number of acoustic clues for the listeners, giving the listener more information to extract. Consequently, the listener can better perceive dysfunctional adjustments made by speakers during phonation^(12,28,60)^. However, in the present study, we also performed auditory-perceptual evaluation of voice quality with all speech tasks interconnected, whose average time ranged from 30 to 34 seconds and which showed a lower interrater reliability.

This decreased interrater reliability can be explained by the single stimulus incorporating different types of speech tasks (sustained vowels and connected speech), which causes variability in the perceptual parameters to be extracted from different speech samples. Hence, the higher the number of parameters of analysis is, the more difficult the rating will be, and the more auditory skills will be required^(1,19,45,57)^. In addition, the rater may focus on a specific segment when analyzing the voice recording, on either the vowel or the connected speech, thus increasing the variation of the data from the auditory-perceptual analysis^(13)^.

Among all speech tasks rated, and even when presenting the stimuli interconnectedly, the task sentences (including six CAPE-V sentences) had the highest interrater reliability coefficient in each study group (specialized speech-language pathologists, generalists, and students with or without experience), as well as substantial reliability (0.75 < ICC < 0.90). Further studies should identify how many CAPE-V sentences together (stimulus duration) are necessary to maintain or improve the level of agreement between listeners in the auditory-perceptual evaluation of voice quality.

In general, interrater reliability was higher when using the VAS than the numerical scale of GRBAS. Previous studies^(6,21,33,34)^ also indicate improved interrater reliability in the auditory-perceptual evaluation of voice quality when using a VAS because the numerical scale, being a categorical scale, is more susceptible to error than the VAS. In addition, the VAS better represents the change of a given deviation and is faster and easier to apply than a numerical scale^(6,21,61)^. Even when using these two scales, the connected speech task CAPE-V sentences had better interrater reliability.

The findings of our study underscore the notion that specific training can increase interrater reliability in the auditory-perceptual evaluation of voice quality. The groups of listeners with the same training showed the best reliability of their ratings. Importantly, the group consisting of general speech-language pathologists, in general, had the best results among all groups. This finding demonstrates that the qualitative (training type) and quantitative (training time) factors of experience, together, may positively affect interrater reliability.

Despite the long experience of SLP-V group (more than ten years), the group was heterogeneous regarding the type of experience of the subjects, in that these professionals worked in different areas of speech-language pathology and had gone through different types of training. Thus, the results from this study indicate that standardized and specialized training with the same reference stimuli may help to improve the interrater reliability of the auditory-perceptual analysis of voice quality.

## CONCLUSION

The time of experience in the auditory-perceptual judgment of the voice, the, measurement scale, and the type of training to which they were submitted, and the type of speech task influence the reliability of the auditory-perceptual evaluation of vocal quality. SLP-NV group present the best interrater reliability, while SLP-V demonstrate the best reliability intrarater in the OS judgment in different speech tasks, using VAS or GRBAS. Regarding the speech task, sentences showed the best interrater reliability coefficients among all tasks and in all groups, when using both the VAS and GRBAS. In general, interrater reliability was higher when using the VAS than when using the GRBAS.
